# Mechanical Properties and Failure Mechanism of Anchored Bedding Rock Material under Impact Loading

**DOI:** 10.3390/ma15196560

**Published:** 2022-09-21

**Authors:** Yunhao Wu, Xuesheng Liu, Yunliang Tan, Qing Ma, Deyuan Fan, Mingjie Yang, Xin Wang, Guoqing Li

**Affiliations:** 1 College of Energy and Mining Engineering, Shandong University of Science and Technology, Qingdao 266590, China; 2 State Key Laboratory of Mining Disaster Prevention and Control Co-Founded by Shandong Province and the Ministry of Science and Technology, Qingdao 266590, China; 3 School of Resources and Environmental Engineering, East China University of Technology, Shanghai 200237, China

**Keywords:** anchored bedding rock material, impact loading, instability mechanism, complex stress environment, shear failure

## Abstract

In view of the problem that anchored bedding rock material is prone to instability and failure under impact loading in the process of deep coal mining, and taking the lower roadway of a deep 2424 coal working face in the Suncun coal mine as the engineering background, a mechanical model of anchored bedding rock material was established, and the instability criterion of compression and shear failure of anchored bedding rock material was obtained. Then, the separated Hopkinson pressure bar was used to carry out an impact-loading test on the anchored bedding rock material, and the dynamic mechanical properties of the rock with different anchoring modes and bolt bedding angles were studied; the evolution law of the strain field of the anchored bedding rock material was also obtained. The results show the following: (1) The bolt support could effectively improve the dynamic load strength and dynamic elastic modulus of the rock material with anchorage bedding, the degree of improvement increased with the increase in the angle of the bolt bedding, and the full anchorage effect was much higher than the end anchorage effect was. (2) The bolt bedding angle and anchorage mode greatly influenced crack development and displacement characteristics. After an impact, the bedding rock material had obvious shear displacement along the bedding direction, and obvious macroscopic cracks were produced in the bedding plane. The research results offer theoretical guidance to and have reference significance for deep roadway anchorage support engineering.

## 1. Introduction

Deep coal resources are an important guarantee as China’s main energy sources [1,2,3]. Statistics show that coal resources with a depth of more than 600 m account for about 73% of the total coal, while coal resources with a depth of more than 1000 m account for about 53%. Deep mining is becoming the new normal in coal-resource development [4,5].

In the process of deep coal mining, the surrounding rocks of deep roadways bear large initial ground stress, and the superposition of supporting pressure leads to stress concentration, which places the surrounding rock in a state of high strength compression for a long time [6,7,8]. In addition, the mining process is often accompanied by the periodic fracturing and the lateral collapse of the overlying strata, fault slips, blasting, and other activities [9,10]. The impact loading generated by these activities causes strong disturbance to the surrounding rock of the roadway [11,12,13,14]. When the static load and impact loading of the surrounding rock in the mining space exceed the critical load of the impact failure of coal and rock material, the surrounding rock becomes unstable, resulting in roof collapses, hydraulic support compression frames, partial sidewalls, and other rock burst accidents [15,16,17,18]. The complex geological environment of the surrounding rock is also an important factor that threatens the stability of the surrounding rock of roadways [19,20]. As layered composite rock material is widely distributed in underground roadways, and the bedding plane of the rock stratum is its natural weak surface, the existence of bedding weakens the antideformation ability of the rock material and reduces the stability of the surrounding rock [21,22,23,24,25]. Therefore, the layered surrounding rock of deep coal mining roadways is prone to instability and failure under impact loading, and supporting it is very difficult [26,27], which has become one of the main bottlenecks restricting the safety of deep coal mining.

Aiming at the failure problem of anchorage structures under impact loading, Wu Yongzheng [28,29] analyzed the dynamic load response characteristics of surrounding anchorage rock under impact loading, and studied the dynamic response law of anchorage rock under lateral impact load, and the influence of impact energy and bolt mechanical properties on rock material mechanical behavior. Mu Zonglong [30] analyzed the failure conditions of roadway surrounding rock under static load, and dynamic and static loads, and experimentally studied the failure conditions of dynamic load on surrounding roadway rock. Wang Zhengyi [31] analyzed the dynamic effect of longitudinal waves, established the criterion of rock burst of anchoring roadway support structures, and formulated the corresponding preventive measures. Wang Aiwen et al. [32] found that there is an obvious time difference effect between the vibration of the bolt and the surrounding rock under impact loads, which leads to the asynchronous vibration of the bolt and the surrounding rock, resulting in the dynamic shear of the anchorage agent. Qiu Penggqi [33] believed that improving the antisliding characteristics and coordinated deformation ability of a rock, the anchoring agent and bolt can effectively prolong the anti-impact aging time of anchoring rock and reduce the impact of dynamic load on the supporting structure of surrounding anchoring rock. Skrzypkowski K [34,35] believed that the places of particular exposure to shear stresses are faults and layered roof layers; between them, there are surfaces of reduced cohesion. Jiao Jiankang [36] analyzed the dynamic load response characteristics and impact failure evolution process of roadway anchorage bearing structures under dynamic load disturbances, and put forward the impact-failure criterion, and the criterion of anchorage bearing structures under dynamic load disturbance and the control technology of surrounding roadway anchorage rock under dynamic load disturbance.

These studies revealed the failure mechanism of anchorage structures under impact loading to a certain extent, and put forward the corresponding control technology of anchorage rocks of rock burst roadways, but failed to fully reveal the overall instability mechanism of anchorage bodies. There is little research on dynamic instability of anchorage bodies under complex stratum conditions, which fails to solve the problem of the instability and failure of anchored bedding rock material that widely exists in engineering geology.

Therefore, the lower roadway of 2424 coal working face in Suncun Coal Mine was taken as the engineering background, the mechanical model of the anchored bedding rock material is established, the compression and shear instability criterion of the anchored bedding rock material is put forward, and the split Hopkinson pressure bar test device was used to carry out an impact loading test on the anchored bedding rock material and reveal the influence law and instability mechanism of the angle between different bolts and bedding and the anchoring mode on the mechanical properties of the bedding rock material.

## 2. Stress Analysis and Instability Mechanism of Anchored Bedding Rock Material

### 2.1. Engineering Background

**Figure 1:**
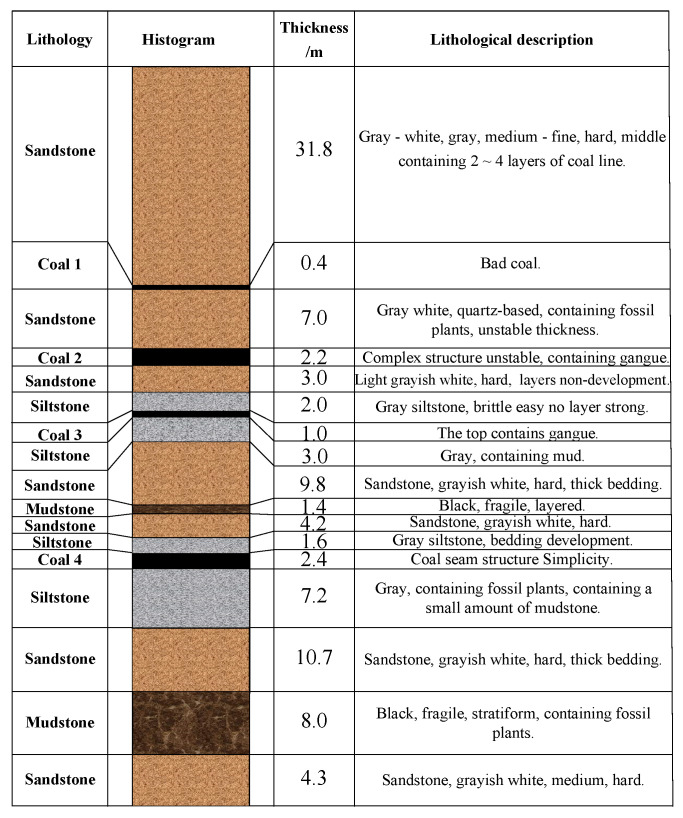
2424 Coal working face rock histogram.

Taking the lower roadway of 2424 coal working face in the Suncun coal mine as an example, the coal seam of the −800 m level 2424 coal working face was four coals, and the rock column diagram is shown in Figure 1.

**Figure 2:**
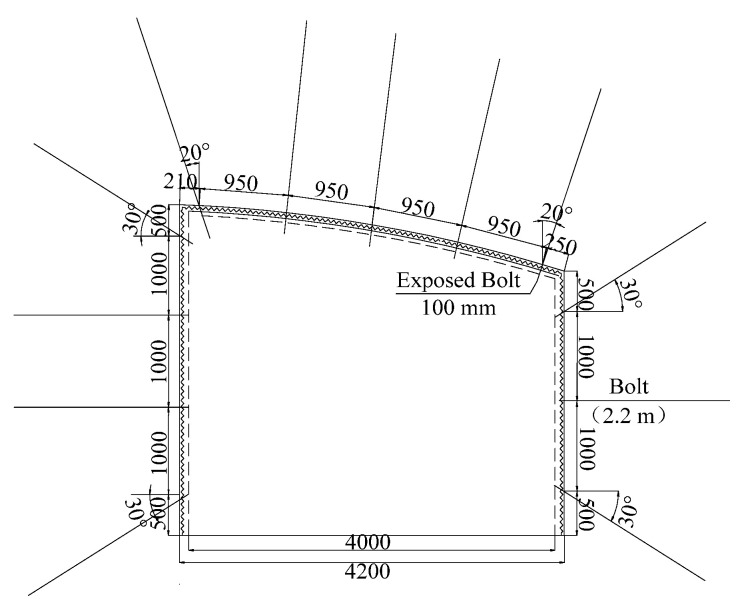
Schematic diagram of roadway support.

The average thickness of the coal seam was 2.4 m, and the dip angle was 26.5°. The direct top was siltstone and sandstone, thickness was about 5.8 m, bedding development was relatively hard, and compressive strength was 21.2 MPa. The basic roof was mainly sandstone with about 11.2 m thick and hard bedding. The buried depth of the lower roadway in the 2424 coal working face was 1254 m, and the total length was 1132 m. The section was trapezoidal, and the net width was 4 m. The height of the left side was 3 m, and the height of the right side was 4 m. The roof was an arc section, as shown in Figure 2.

The mining process of the coal working face uses a Φ22 × 2400 mm HRB 600 resin anchored anchor bolt + diamond mesh + W steel belt + tray support. The anchor bolt anchorage length was 1.2 m. The three middle rows of anchor bolts were installed on the vertical roof (at an angle of 14° with the vertical line), and the outermost anchor bolts were installed at an angle of 20° with the vertical line.

This was adopted by two gangs, namely, Φ22-2200 mm MSGLD-600(x) equal strength threaded steel bolt + diamond mesh + W steel strip + high-strength tray support. The lowest row of bolts on the two sides of the roadway was 500 mm from the roadway floor and inclined downward at an angle of 30° with the horizontal line. The lowest row of bolts on the two sides of the roadway was 500 mm from the roadway roof and inclined upward at an angle of 30° with the horizontal line.

A half-length anchor was adopted for the two sides and the roof. The anchoring length was 1.4 m. Fast-setting resin was selected as the anchoring agent, composed of two kinds of materials, namely, resin cement (cement containing resin, dolomite powder, silica, and accelerator) and a curing agent (containing benzoyl peroxide, light calcium, and water).

### 2.2. Stress Analysis of the Anchored Rock Material

The dynamic action in the mining process mainly comes from mining activity and the stress response of surrounding rock to the mining activity. The mining process of a coal seam is always accompanied by the movement of the overlying strata. The high stress waves generated by the roof breaking in the movement of the overlying strata form mutual interference and superposition in the coal and rock, and have a strong disturbance effect on the surrounding rock of a roadway.

**Figure 3:**
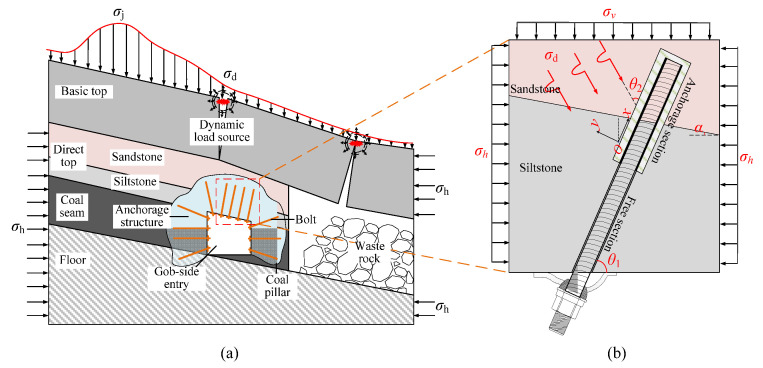
Stress environment of the surrounding rock and mechanical model of anchored bedding rock material. (**a**) Anchorage structure and stress environment of layered composite surrounding rock in Inclined Mining Roadway; (**b**) Mechanical model of anchorage bedding rock mass.

Taking the lower drift of coal working face 2424 of the Suncun coal mine as an example, when the layered composite surrounding rock roof and two sides of the deep mining roadway were supported by bolts, the anchored surrounding rock was subject to high ground stress and impact loading, which led to the surrounding anchored layered rock very easily losing stability and breaking the ring, as shown in Figure 3a.

In order to study the stress condition and instability mechanism of the anchored surrounding rock at the bedding development under impact loading, the mechanical model of the anchor under impact loading was established with the roof anchor as the research object, as shown in Figure 3b. The inclination angle of the anchor rod was *θ*_1_, and bedding dip angle was *α*. The anchor body was subjected to vertical stress of surrounding rock *σ*_v,_ and horizontal stress *σ*_h_ was also affected by the dynamic load disturbance caused by roof fracture instability. The dynamic load was *σ*_d_, which included angle with anchor rod *θ*_2_.

A dynamic load generated by roof fracture instability mainly propagates in the form of stress waves in the rock structure. As the surrounding rock in the actual project is heterogeneous and anisotropic material, it is difficult to accurately analyze it. Therefore, the following were assumed: ① The surrounding rock of the roadway is an ideal homogeneous elastic–plastic body; ② at a certain distance from the source, the stress waves propagating in the rock can be regarded as plane waves [37]. The shear wave effect of the stress waves was not considered, and the dynamic load (*σ*_p_) generated by longitudinal waves (P) P can be expressed as follows:

(1) $$\sigma_{p}=\rho C_{p}v_{p}$$

where *C*_p_ is the propagation velocity of P waves, m/s; *v*_p_ is the peak vibration velocity of the particle at the boundary of the dynamic load source, m/s; and *ρ* is the density of rock mass, kg/m^3^.

The research shows that the propagation of stress waves in a coal rock medium presents a power function attenuation law [38], and the attenuation law of particle vibration velocity can be expressed as follows:

*v* = *v*_p_*L*^−*η*^ (2)

where *v* is the peak vibration velocity of the particle at the propagation of the stress wave, m/s; *L* is the distance from the boundary of a moving source, m; *η* is the peak velocity attenuation coefficient related to the propagation medium; attenuation coefficients *η* of the intact rock mass, intact coal body, and fractured coal body are [38] 0.5755, 0.867, 1.0655, respectively.

Combining Formulas (1) and (2), the dynamic load caused by stress-wave propagation to the surface of the anchorage structure is:

(3) $$\sigma_{d}=\rho C_{p}v_{p}L^{-\eta }$$

At this time, stress at any point O on the anchorage body can be characterized along the inclined direction (x axis) and the vertical direction (y axis) of the bolt as follows:

(4) $$\left\{\begin{array}{l} \sigma_{1}=\sigma_{x}=\sigma_{v}\cdot \sin\theta_{1}+\rho C_{p}v_{p}L^{-\eta }\cdot \cos\theta_{2}\\ \sigma_{3}=\sigma_{y}=\sigma_{v}\cdot \cos\theta_{1}+\rho C_{p}v_{p}L^{-\eta }\cdot \sin\theta_{2} \end{array}\right.$$

According to the Mohr–Coulomb criterion, because of *σ*_3_ > 0, when $\sigma_{1}=\sigma_{v}\cdot \sin\theta_{1}+\rho C_{p}v_{p}L^{-\eta }\cdot \cos\theta_{2}\geq \sigma_{c}$ (*σ*_c_ is uniaxial compressive strength of anchorage), compression fracture occurs in the surrounding rock of an anchor solid.

### 2.3. Shear Failure Mechanism of Anchorage Rock Material

**Figure 4:**
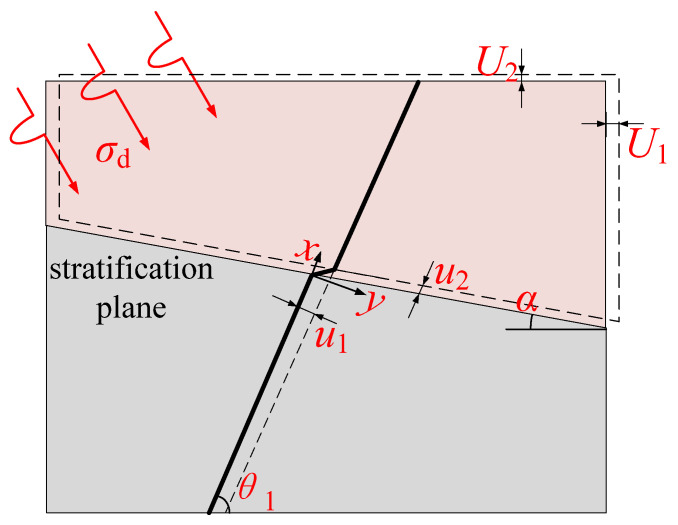
Instantaneous shear model of anchor solid bedding plane.

Since the dynamic load propagation process is transferred from sandstone to siltstone, and the propagation velocity of stress waves in different strata is different, shear dislocation occurs on the joint surface, and shear failure occurs on the anchorage body. In order to analyze this problem, the instantaneous shear model of anchor solid bedding surface under dynamic load was established, as shown in Figure 4. Assuming that the transverse displacement of the structural plane was *U*_1_, the longitudinal displacement was *U*_2_, and *U*_2_ = *U*_1_ tan*ψ*, *ψ* is the shear angle of the structural plane.

Then, the lateral deformation of the bolt is *u*_1_, and the axial deformation is *u*_2_. According to the geometric relationship:

(5) $$\begin{array}{l} u_{1}=U_{1}\sin\left(\alpha +\theta_{1}\right)/\cos\;\alpha \\ u_{2}=U_{2}/\sin\left(\alpha +\theta_{1}\right) \end{array}$$

The shear strength of the anchored bedding rock material is generally composed of four parts: the shear strength of the bedding plane itself *τ_j_*, the shear strength contributed by the ‘pin’ action of the bolt body *τ_bd_*, and the shear strength contributed by the normal and tangential components of the axial load of the bolt along the joint surface *τ_bd_*, *τ_bs_* [39]. Therefore, shear strength *τ_bd_* of the anchored bedding rock material can be expressed as follows:

(6) $$\tau_{bj}=\tau_{j}+\tau_{bd}+\tau_{bt}+\tau_{bs}$$

According to the Coulomb–Mohr criterion, the shear strength *τ_j_* of the bedding plane itself is:

(7) $$\tau_{j}=\sigma_{j}\tan\left(\psi +\varphi^{\prime }\right)+c_{j}$$

where *σ_j_* is the normal stress of the structural plane, *c_j_* is the structural surface adhesion, and $\varphi^{\prime }$ is the structural surface friction angle.

*τ_bd_*, *τ_bd_*, and *τ_bd_* can be calculated with the following formula:

(8) $$\left\{\begin{array}{l} \tau_{bd}=\tau_{b}\cdot \eta \left[\sin\left(\alpha +\theta_{1}\right)/\cos\;\alpha \right]\\ \tau_{bt}=\sigma_{b}\cdot \sin\left(\alpha +\theta_{1}\right)\cdot \eta \cdot \tan\varphi^{\prime }\\ \tau_{bs}=\sigma_{b}\cdot \cos\left(\alpha +\theta_{1}\right)\cdot \eta \end{array}\right.$$

where *σ_b_* is axial stress of the bolt, *τ_b_* is the average shear stress on the cross-section of the bolt, and *η* is the ratio of the cross-sectional area of the bolt to the area of the bedding plane containing a single bolt.

**Figure 5:**
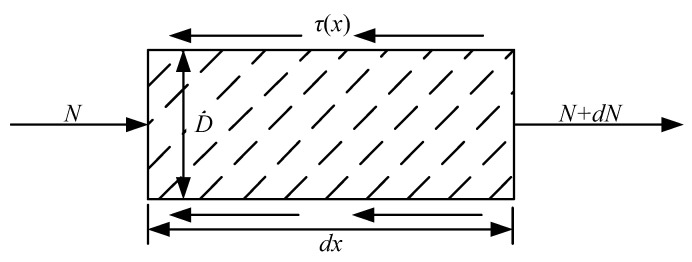
Mechanical model of a bolt element.

In order to obtain the axial stress of the bolt, a bolt element was taken for stress analysis, as shown in Figure 5.

According to the unit force balance, the differential equation is established:

(9) $$N\left(x\right)-\pi D\tau \left(x\right)dx=N\left(x\right)+dN\left(x\right)$$

where *N*(*x*) is the axial force of the bolt at position *x* from the bedding plane, *D* is the bolt diameter, and *τ*(*x*) is the interfacial shear stress at *x* position from the bedding plane.

Through the linear relationship between load and displacement, and the stress equilibrium state of bolt, the equation of interfacial shear stress with displacement is obtained:

(10) $$\tau \left(x\right)=Ku\left(x\right)$$

where *u*(*x*) is the axial normal strain of the bolt; and *K* is the comprehensive shear stiffness, $K=\frac{K_{1}K_{2}}{K_{1}+K_{2}}$(*K*_1_ and *K*_2_ are shear stiffness of slurry and shear stiffness of rock material, respectively).

Combining Formulas (9) and (10), we can obtain the following:

(11) $$\frac{dN\left(x\right)}{dx}=\pi DKu\left(x\right)$$

Then, the axial strain *ε*(*x*) of the bolt at a distance from the bedding plane *x* can be expressed as follows:

(12) $$\varepsilon \left(x\right)=\frac{du\left(x\right)}{dx}=\frac{\sigma_{x}}{E}=\frac{N\left(x\right)}{EA}$$

where *E* is the elastic modulus of the anchor, and *A* is the cross-sectional area of the anchor.

From the above analysis:

(13) $$\frac{d^{2}N\left(x\right)}{dx^{2}}-\frac{K\cdot N\left(x\right)}{EA}=0$$

When the bedding plane is damaged, it is assumed that the axial force of the bolt at the bedding plane is pretightening force *N*_0_, and the axial force at the distance from the joint plane *u*_2_ (axial deformation of the bolt) is 0. Therefore, the boundary conditions are *N* (*x* = 0) = *N*_0_, *N* (*x* = *l*) = 0, and the solution is:

(14) $$N\left(x\right)=N_{0}\frac{\sin h\left[\sqrt{\frac{K}{EA}}\left(u_{2}-x\right)\right]}{\sin\left(u_{2}\sqrt{\frac{K}{EA}}\right)}$$

Then the axial stress *σ_b_* of bolt is:

(15) $$\sigma_{b}=\frac{N_{0}\sin h\left[\sqrt{\frac{4K}{E\pi D^{2}}}\left(u_{2}-x\right)\right]}{\frac{\pi D^{2}}{4}\sin\left(u_{2}\sqrt{\frac{4K}{E\pi D^{2}}}\right)}$$

Before the failure of the bedding anchor solid, the anchor and rock near the bedding plane are in an elastic state. According to the principle of elastic mechanics, the lateral deformation of the anchor satisfies:

(16) $$\frac{d^{4}u_{1}}{dy^{4}}+\frac{Dk}{EI}u_{1}=0$$

where *k* = 300 *σ*_c_/*D*, the rock reaction coefficient; and *EI* is the anchor bending stiffness.

Analysis shows that, when *y* = 0, the bending moment is 0; when *y*→∞, the bending moment is 0, and the shear force is 0:

(17) $$\left\{\begin{array}{l} M_{y=0}=-EI\frac{d^{2}u_{1}}{dy^{2}}=0\\ M_{y\to \infty }=-EI\frac{d^{2}u_{1}}{dy^{2}}=0\\ \tau_{y\to \infty }=-EI\frac{d^{3}u_{1}}{dy^{3}}=0 \end{array}\right.$$

From these boundary conditions, the relationship between the lateral displacement of the bolt and shear stress *τ_b_* in the shear-slip process of the bedding plane can be obtained:

(18) $$\tau_{b}=\frac{8EIu_{1}\left(y\right)}{pD^{2}{\left(\frac{4EI}{kD}\right)}^{\frac{3}{4}}}$$

The reaction force provided by the rock in the elastic state is *p* = *ku*_1_. When the compressive stress is too large, the rock produces plastic failure and reaches the ultimate reaction, *p* = *nσ*_c_. At this time, the lateral elastic displacement limit of the bolt end is *u*_1_ = *nD*/300. *n* is a coefficient dependent on the internal friction angle of rock, and its value is 2–5 according to the degree of rock hardness.

Comprehensive Formulas (6)–(8), (15) and (18) can be substituted into numerical values to obtain the shear strength at any point (*x*, *y*) of the rock material with anchor bedding:

(19) $$\begin{array}{l} \tau_{bj}\left(x,y\right)=\sigma_{j}\tan\left(\psi +\varphi^{\prime }\right)+\frac{N_{0}\sin h\left[\sqrt{\frac{4K}{E\pi D^{2}}}\left(u_{2}-x\right)\right]}{\frac{\pi D^{2}}{4}\sin\left(u_{2}\sqrt{\frac{4K}{E\pi D^{2}}}\right)}\cdot \eta \left[\sin\left(\alpha +\theta_{1}\right)/\cos\;\alpha \right]+\\ \frac{8EIu_{1}\left(y\right)\eta }{pD^{2}{\left(\frac{4EI}{kD}\right)}^{\frac{3}{4}}}\cdot \left[\sin\left(\alpha +\theta_{1}\right)\cdot \tan\varphi^{\prime }+\cos\left(\alpha +\theta_{1}\right)\right] \end{array}$$

When $\sigma_{d}\sin\left(\theta_{1}+\alpha \right)+\sigma_{v}\sin\alpha =\rho C_{p}v_{p}L^{-\eta }\sin\left(\theta_{1}+\alpha \right)+\sigma_{v}\sin\alpha \geq \tau_{bj\max}$, the bedding plane shear failure occurs in the anchored bedding rock material under dynamic load disturbance, resulting in anchoring failure.

On the basis of the stress analysis of anchored bedding rock material, the instability criterion of anchored bedding rock material in a complex stress environment can be obtained:

(20) $$\left\{\begin{array}{l} \sigma_{v}\cdot \sin\theta_{1}+\rho C_{p}v_{p}L^{-\eta }\cdot \cos\theta_{2}\geq \sigma_{c},\;\mathrm{Compression}\;\mathrm{Failure}\\ \rho C_{p}v_{p}L^{-\eta }\sin\left(\theta_{1}+\alpha \right)+\sigma_{v}\sin\alpha \geq \tau_{bj\max},\;\mathrm{Shear}\;\mathrm{Failure} \end{array}\right.$$

where *σ*_c_ is the uniaxial compressive strength of rock mass.

According to this formula, the parameters of the bedding anchor solid can be obtained with field measurements, and physical and mechanical tests, and the instability of the anchored bedding rock material can be judged:

① When $\sigma_{v}\sin\theta_{1}+\rho C_{p}v_{p}L^{-\eta }\cdot \cos\theta_{2}\geq \sigma_{c},\;\rho C_{p}v_{p}L^{-\eta }\sin\left(\theta_{1}+\alpha \right)+\sigma_{v}\sin\alpha \geq \tau_{bj\max}$, shear compression failure occurred in the anchored bedding rock material.
② When $\sigma_{v}\sin\theta_{1}+\rho C_{p}v_{p}L^{-\eta }\cdot \cos\theta_{2}<\sigma_{c},\;\rho C_{p}v_{p}L^{-\eta }\sin\left(\theta_{1}+\alpha \right)+\sigma_{v}\sin\alpha \geq \tau_{bj\max}$, shear failure occurred in the anchored bedding rock material.
③ When $\sigma_{v}\sin\theta_{1}+\rho C_{p}v_{p}L^{-\eta }\cdot \cos\theta_{2}\geq \sigma_{c},\;\rho C_{p}v_{p}L^{-\eta }\sin\left(\theta_{1}+\alpha \right)+\sigma_{v}\sin\alpha <\tau_{bj\max}$, compression failure of the anchored bedding rock material.
④ When $\sigma_{v}\sin\theta_{1}+\rho C_{p}v_{p}L^{-\eta }\cdot \cos\theta_{2}<\sigma_{c},\;\rho C_{p}v_{p}L^{-\eta }\sin\left(\theta_{1}+\alpha \right)+\sigma_{v}\sin\alpha <\tau_{bj\max}$, the anchored bedding rock material is not damaged.

## 3. Anchored Bedding Rock Material Impact Loading Test

### 3.1. Sample Preparation

According to the instability criterion of anchored bedding rock material, under the condition of a constant surrounding rock stress environment, the angle between bolt and bedding, and the physical and mechanical properties of anchorage body are important factors affecting the instability of anchored bedding rock material. In order to further study the mechanical properties and instability mechanism of anchored bedding rock material, the impact loading test of anchored bedding rock material was carried out.

**Figure 6:**
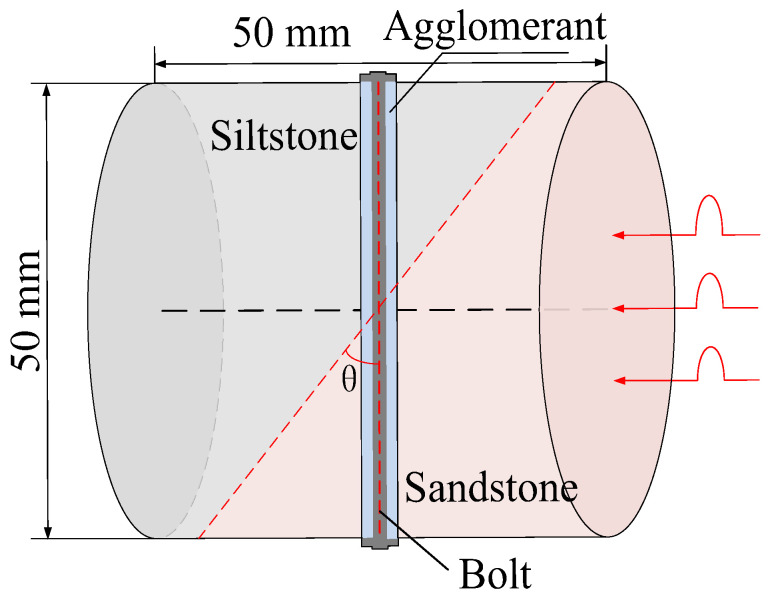
Schematic diagram of inclined bedding surrounding rock anchorage specimen.

According to the stress conditions of the anchor solid, as shown in Figure 3b, assuming that the dynamic load of the rock fracture acted vertically on the anchor, the anchorage bedding rock specimen was produced as shown in Figure 6, where the angle between the anchor and the bedding plane was *θ*, *θ* = *α* + *θ*_1_.

Taking the surrounding rock of the lower roadway in the 2424 working face of the Suncun coal mine as the research object, the siltstone and sandstone of the roof were selected. After drilling, cutting, and grinding, columnar specimens with different inclination angles were produced. The siltstone and sandstone specimens were bonded with epoxy resin, and specimens of Φ50 × *h* 50 mm were produced. In order to study the influence of the bolt bedding angle and anchorage method on the mechanical properties of anchorage rock material, three groups of specimens were designed. As shown in Table 1, Group A was the control group and the full-length anchorage. The bolt bedding angle was 15°. Test Group 1 was anchorage specimens with different bolt bedding angles, and the bolt bedding angles were 30° and 45°, which were all full-length anchorage specimens. Test Group 2 was the anchorage specimens with different anchorage modes, and the angle between the bolt beddings was 15°. The anchorage modes were nonanchor and end anchor.

The anchoring process of the full-anchor specimen was as follows. First, a 5 mm drilling rig was used to drill axially into the middle of the side of the composite specimen. A 4 mm high-strength rebar was used as the indoor experimental anchor, and a flat cushion was used to simulate the tray. A mechanical torque wrench was used to impose preload torque on the anchor. Then, an epoxy resin adhesive was selected as the anchoring agent and was injected into the pores with a needle pipe. After a period of time, it solidified. The end-anchored specimen only used high-strength rebar as the anchor rod, and the flat pad simulated the tray. After applying the preload torque, no anchoring agent was added. The size parameters and preload torque are shown in Table 2.

After the preparation of each type of specimen had been completed, the strain gauge (SG1) was pasted perpendicular to the loading direction at the center of the specimen disk surface to monitor the strain of the anchor solid matrix during the test. The strain gauge (SG2) was pasted onto the middle surface of the bolt to monitor the tensile strain of the bolt during the test.

### 3.2. Testing Equipment

**Figure 7:**
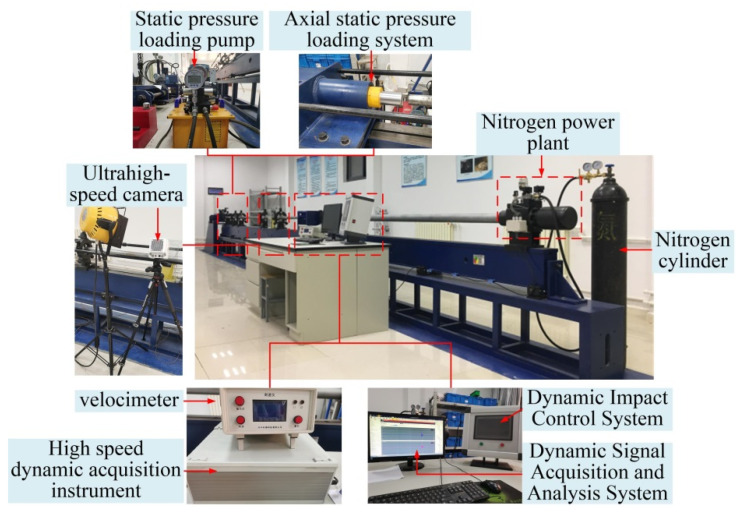
Separation Hopkinson pressure bar test system.

The test equipment comprised the Φ50 mm split Hopkinson pressure bar (SHPB) system, as shown in Figure 7. The incident rod, transmission rod, and absorption rod are produced with a Φ50 mm steel rod with an elastic modulus of 206 GPa, and the compression rod material was 48CrMoA. The length of the man-shot and transmission rods was 1500 mm, and the length of the absorption rod was 1000 mm. In order to produce sine waves with a slow loading section, we adopted a spindle-shaped bullet of the same material for the punch with a maximal diameter of 50 mm and length of 365 mm, and the strain signal transmission frequency and maximal sampling frequency of the dynamic strain gauge were 1 MHz.

### 3.3. Test Scheme

Before the test, the front and rear faces of the specimen were ground, polished, and smeared with lubricant to ensure good contact between the specimen, and the input and output rods. Speckles were produced on the surface of the specimen in advance. During the test, a VisionResearch/V410L high-speed camera was used to capture the whole process of the impact failure of the specimen, and the digital image correlation method was used to obtain the crack and displacement changes during the failure of the specimen.

During the impact loading process of the rock material with anchorage bedding, the designed stress wave was incident from the vertical bolt on the side of sandstone, and the composite specimen without anchorage bedding was tested several times to determine the appropriate impact velocity and ensure the complete fracture of the specimen; the impact speed of each group of test pieces is shown in Table 2. After the preparation work had been completed, the impact loading test of the anchored bedding rock material was carried out successively. The incident and reflected waves, and transmission pulse in the transmission rod were measured and recorded with the strain gauge. According to [36], the dynamic strain *ε*(*t*), strain rate $\dot{\varepsilon }$, and stress *σ* of the sample are indirectly obtained by using the three-wave method. The calculation formula is as follows:

(21) $$\varepsilon \left(t\right)=c_{0}/l_{0}\int_{0}^{t}\varepsilon_{i}-\varepsilon_{r}-\varepsilon_{t}d\tau$$

(22) $$\dot{\varepsilon }=c_{0}/l_{0}\left(\varepsilon_{i}-\varepsilon_{r}-\varepsilon_{t}\right)$$

(23) $$\sigma =AE/2A_{0}\left(\varepsilon_{i}+\varepsilon_{r}+\varepsilon_{t}\right)$$

where *c*_0_ is the elastic wave velocity of the bar, m/s; *A*_0_ is the original cross-sectional area, m^2^; *ε_i_*, *ε_r,_* and *ε_t_* are the time-history strains of the incident wave, reflected wave, and transmitted wave when they propagated independently; *A* is the cross-sectional area of the rod, m^2^; and *E* is the elastic modulus of the rod, Gpa.

## 4. Test Result Analysis

### 4.1. Characteristics of Stress–Strain Curve

**Figure 8:**
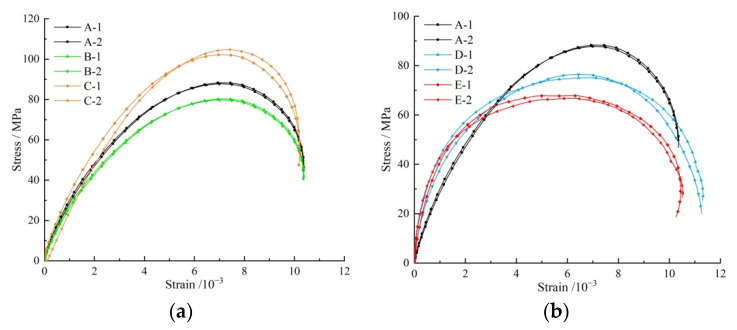
Stress–strain curve. (**a**) Angle between bolt and bedding; (**b**) Anchoring method.

In order to obtain the influence law of bolt bedding angle and anchorage mode on the mechanical properties of the anchored bedding rock material, the stress–strain curve characteristics of the specimen were first analyzed. In the test, all specimens were subjected to compressive shear failure under impact loading, and the fracture was relatively complete. The dynamic stress–strain curves of the specimens are shown in Figure 8. The experimental results are shown in Table 3.

Figure 8 and Table 3 show that all specimens had slight crack compaction signs at the initial loading stage and then quickly entered the elastic state; stress and strain were basically linearly increased, and then the stress increase tended to be flat. When the stress reached the peak, the rock was destroyed, and the stress dropped to the plastic state. The angle between bolt and bedding, and anchorage mode affected the dynamic stress–strain characteristics of the anchor solid.

The analysis of Experimental Group 1 shows that the average peak stress of the specimens (C-1, C-2) with an angle of 45° was 103.47 MPa, 17.57% higher than that of the specimen with 30° (B-1, B-2), and 29.31% higher than that of the specimen with 15° (A-1, A-2). The dynamic load strength of anchorage body increased with the increase in the angle between anchor and bedding, but the influence is limited. The analysis of Experimental Group 2 shows that the average peak stress of the fully anchored specimen (A-1, A-2) was 80.02 MPa, which is 17.42% higher than that of the end-anchored specimen (D-1, D-2), and 32.16% higher than that of the nonanchored specimen. The bolt support could effectively improve the dynamic load strength of the inclined bedding rock material, and the fully anchored effect was much higher than that of the end-anchored effect.

### 4.2. Dynamic Elastic Modulus of the Specimen

In order to investigate the influence of different bedding angles and anchorage modes on the dynamic deformation capacity of anchorage body, the dynamic elastic modulus *E_d_* of the specimen was calculated according to Formula (24).

(24) $$E_{d}=\frac{2P\left(t\right)}{\pi DL}\frac{2D\left[1-0.7854\left(1-\mu \right)\right]}{d_{ox}}$$

where *P*(t) is the failure load, *μ* is Poisson’s ratio of the rock mass before peak stress, *d_ox_* is the total displacement of the specimen center along the diameter direction in the vertical loading direction when the specimen is damaged, *D* is the specimen diameter, and *L* is the length of specimen.

**Figure 9:**
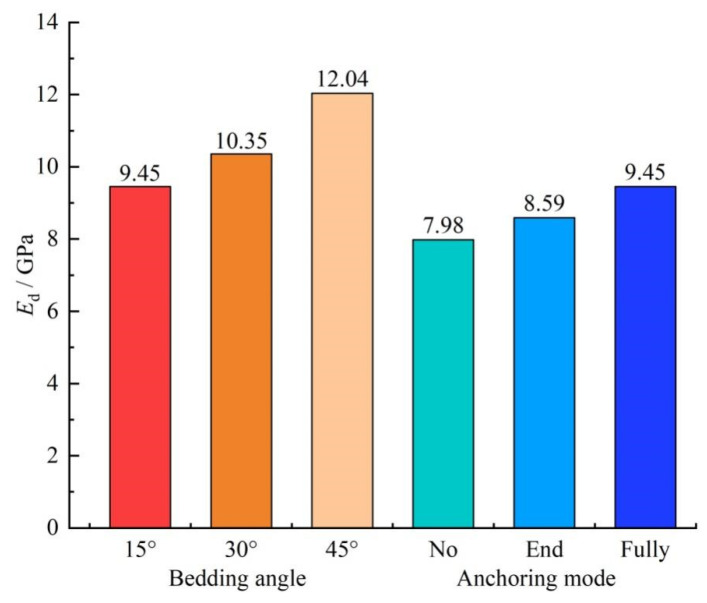
Dynamic elastic modulus (*E_d_*).

Dynamic elastic modulus *E_d_* under different conditions was obtained with numerical calculation, as shown in Figure 9.

Figure 9 shows that the angle between the bolt and bedding, and the anchoring mode impacted the dynamic elastic modulus of the specimen. The *E_d_* of the specimen increased with the increase in the angle between the bolt and bedding, and the increase was obvious. When the angle was 15°, the *E_d_* of the specimen was 9.45 GPa. When the angle was 30° and 45°, the *E_d_* of the anchorage body increased by 9.52% and 27.41%, reaching 10.35 and 12.04 GPa, respectively, and the tensile elastic modulus was effectively improved. Analysis shows that, with the increase in the angle between the bolt and the bedding, when it was subjected to dynamic load impact, macroscopic crack propagation is difficult, and it is more difficult to damage the rock material, so that the dynamic elastic modulus of the anchorage bedding specimen is improved.

In the nonanchor, the dynamic elastic modulus *E_d_* was 7.98 GPa. After the end anchorage, the *E_d_* of the specimen was improved to a certain extent, about 7.64%, reaching 8.59 GPa. When the full anchor was used, the *E_d_* of the specimen was increased by 18.42% to 9.45 GPa, and the tensile elastic modulus of the specimen was significantly improved. Analysis shows that, under dynamic load impact, the bolt of the end-anchored specimen had an axial anchoring effect that changed the tensile properties of the anchorage body. For the fully anchored specimen, the bolt also had an axial and tangential anchoring effect, thus effectively improving its dynamic elastic modulus.

### 4.3. Evolution Law of Strain Field

**Figure 10:**
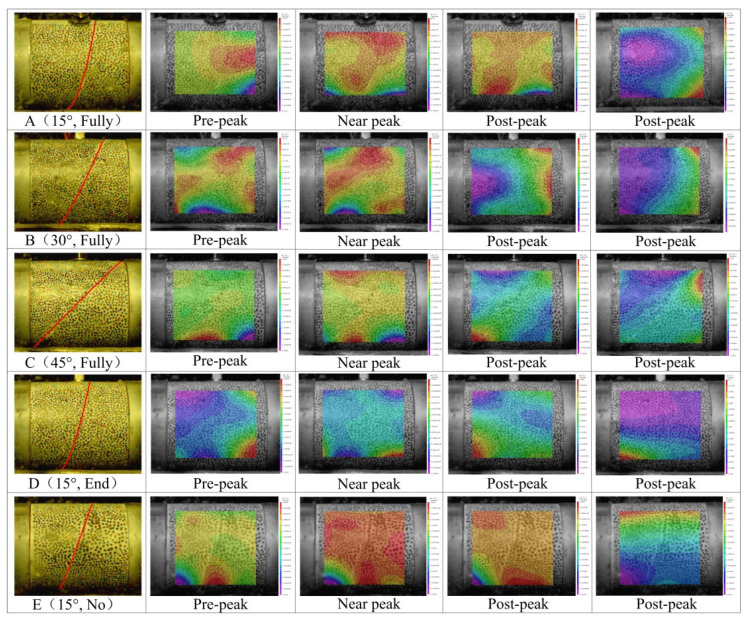
y-direction nephogram of the dynamic load response of an anchorage body.

In order to explore the strain field evolution law of an anchored bedding rock mass under impact dynamic load, and to study the shear displacement process of a rock mass along the bedding direction, an ultrahigh-speed camera and digital speckle measurement technology were used during the test [33,39]. By capturing the y-direction strain cloud map of the dynamic load response of the anchored bedding rock mass from prepeak to postpeak under dynamic load, the strain field evolution law of anchored bedding rock mass was obtained. The results are shown in Figure 10.

Figure 10 shows that, after the full-anchor specimen with an angle of 15° between the bolt and the bedding had been subjected to impact, the central position of the impact side first formed the maximal y-direction strain concentration zone and gradually extended inward. When it extended to the vicinity of the bedding plane, the strain began to expand along the bedding face to both sides of the tip, producing macroscopic cracks and gradually extending to the end of the specimen. At that time, when the specimen was in the peak state, the failure of the specimen entered the plastic state, and the crack propagation of the bedding plane gradually stopped. The anchorage rock material formed a more obvious shear movement along the vicinity of the bedding plane, and the coarse sandstone of the impact side moved upward, while the strain of the fine sandstone side was smaller and more stable. After the full-anchor specimen with an angle of 30° had been impacted, the maximal y-direction strain concentration zone first formed near the central position of the impact side and the upper tip of the bedding plane, and gradually extended inward; then, the maximal strain zone began to expand along the bedding plane towards the central area, resulting in macroscopic cracks. The specimen broke into the postpeak plastic stage, and the crack propagation of the bedding plane gradually stopped. The anchorage rock material formed a more obvious shear movement near the bedding plane, and the coarse sandstone moved upward on the impact side, while the strain of the fine sandstone side was small and stable. After the full-anchor specimen with an angle of 45° had been impacted, the maximal y-direction strain concentration zone was first formed near the bottom bedding plane of the specimen, and the maximal strain zone began to expand along the bedding plane to the upper region. The strain near the bedding plane was much larger than that on both sides. The macroscopic cracks of the through specimen were generated at the bedding plane. Subsequently, the specimen broke into the postpeak plastic stage, and the fracture expansion of the bedding plane gradually stopped. The anchoring rock material formed a relatively obvious shear movement near the bedding plane. The coarse sandstone on the impact side moved upward at an angle of 45°, while the strain on the fine sandstone side was small and stable.

At the same time, Figure 9 also shows that, after the end-anchored specimen had been impacted, the maximal y-direction strain area formed by the impact side gradually expanded inward, and the strain near the joint surface was slightly higher than that in other areas. After entering the postpeak stage, the upper part of the specimen was affected by the end-anchored anchorage, and its strain was small, which is in sharp contrast to the free section of the lower part. In addition, the overall strain of the specimen was higher than that of the fully anchored specimen, and the fracture characteristics were more obvious.

At the same time, Figure 9 also shows that, after the end-anchored specimen had been impacted, the maximal y-direction strain area formed by the impact side gradually expanded inward, and the strain near the joint surface was slightly higher than that in other areas. After entering the postpeak stage, the upper part of the specimen was affected by the end-anchored anchorage, and its strain was small, which is in sharp contrast to the free section of the lower part. In addition, the overall strain of the specimen was higher than that of the fully anchored specimen, and the fracture characteristics were more obvious. After the impact on the nonanchor specimen, the maximal y-direction strain region of the impact side gradually extended inward. When it extended to the vicinity of the joint surface, the right surrounding rock showed obvious upward displacement compared with the left surrounding rock. With the further evolution of the strain field, the overall displacement of the unanchored specimen appeared to be larger, the strain near the joint was larger, and an obvious shear motion was formed along the joint surface. When it entered the postpeak stage, the shear motion gradually stopped, but the overall displacement of the specimen was still much larger than that of the end- and full-anchor specimens.

In summary, under impact loading, there were obvious differences in the crack development and displacement characteristics of the rock material with different angles and anchorage modes of bolt and bedding. After the impact, the bedding rock material had obvious shear displacement along the bedding direction, obvious macroscopic cracks appeared on the bedding surface, and failure and instability occurred; with end-anchored and nonanchor support, the overall displacement of the specimen was significantly increased, and the fracture characteristics were also more obvious.

Therefore, the anchored bedding rock material was prone to shear failure due to the impact loading. In field engineering practice, it is still necessary to be vigilant about the shear failure of anchored bedding rock material and compression failure. In practice, we could determine the comprehensive shear strength and uniaxial compression strength of the anchored bedding rock material through the impact loading test. After obtaining the stress environment of the surrounding rock and analyzing the stress state of the anchorage body, the strength of the anchored bedding rock material was checked, and the corresponding measures were adopted to ensure that the failure and instability of the anchoring surrounding rock did not occur.

## 5. Conclusions

(1) On the basis of the Coulomb–Mohr criterion and stress propagation theory, the mechanical model of the anchored bedding rock material was established, and the instability criterion of anchored bedding rock material under impact loading was obtained:

$$\left\{\begin{array}{l} \sigma_{v}\cdot \sin\theta_{1}+\rho C_{p}v_{p}L^{-\eta }\cdot \cos\theta_{2}\geq \sigma_{c},\;\mathrm{Compression}\;\mathrm{Failure}\\ \rho C_{p}v_{p}L^{-\eta }\sin\left(\theta_{1}+\alpha \right)+\sigma_{v}\sin\alpha \geq \tau_{bj\max},\;\mathrm{Shear}\;\mathrm{Failure} \end{array}\right.$$

On the basis of this criterion, the stress environment of the surrounding rock, and the mechanical parameters of the rock material obtained from field and test could be used to determine whether the anchored bedding rock material would fail and the failure type.

(2) All specimens were subjected to compression shear failure under impact loading, and the dynamic load strength of the anchored bedding rock material increased with the increase in the angle between the bolt and the bedding. When the angle increased from 15° to 45°, the dynamic load strength of the anchoring solid increased by 29.31%. In addition, bolt support could effectively improve the dynamic load strength of the rock material with anchorage bedding, and the full-length anchor effect was much higher than the end-anchor effect.
(3) With the increase in the angle between the bolt and the bedding, when it was impacted by dynamic load, the macroscopic crack propagation was more difficult, and the rock material was more difficult to damage. The dynamic elastic modulus of the anchoring bedding specimen was improved. When the angle increased from 15° to 45°, the elastic modulus of the anchoring solid increased by 27.41%. Under impact loading, the axial anchoring effect of the end-anchored specimen changed the tensile properties of the anchorage body. For the full-length anchor specimen, the bolt also had an axial and tangential anchoring effect, thus effectively improving its dynamic elastic modulus.
(4) Under impact loading, there were obvious differences in the crack development and displacement characteristics of rock material with different anchoring methods and angles between bolt and bedding. After impact, the bedding rock material had obvious shear displacement along the bedding direction, obvious macroscopic cracks were produced on the bedding plane, and failure and instability occurred. With end-anchored and nonanchored support, the overall displacement of the specimen was significantly increased, and the fracture characteristics were also more obvious.

## Figures and Tables

**Table 1:** Anchored bedding rock material impact loading test design table.

Group	Schematic Diagram 1	Schematic Diagram 2	Schematic Diagram 3
Test 1: bedding angle.	A: 15°	B: 30°	C: 45°
	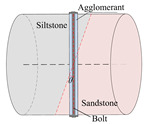	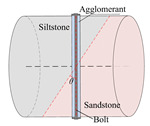	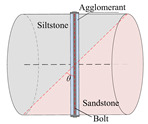
Test 2: anchored form.	A: full-length anchor.	D: end anchor.	E: nonanchor.
	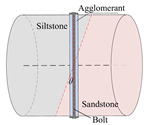	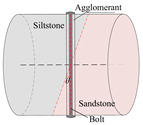	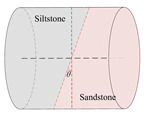

**Table 2:** Test specimen parameters.

Number	Anchored Form	Bedding Angle/°	Tightening Torque/N·m	Diameter/mm	Height/mm	Dynamic Speed/m/s
A-1 A-2	Full-length anchor	15	19.9 20.1	49.9 50.0	49.9 50.0	9.24 9.21
B-1 B-2	Full-length anchor	30	20.0 20.0	50.0 50.0	49.9 50.0	9.27 9.22
C-1 C-2	Full-length anchor	45	19.9 20.0	50.0 50.1	50.0 50.0	9.23 9.20
D-1 D-2	End anchor	15	20.1 20.0	49.9 50.0	50.0 50.0	9.19 9.22
E-1 E-2	Nonanchor	15	0 0	50.0 49.9	49.9 50.0	9.25 9.28

**Table 3:** Dynamic strength and peak strain of anchorage rock specimens.

Groups	Number and Category		Peak Stress/MPa	Mean Peak Stress/MPa	Peak Strain/10^−3^	Mean Peak Strain/10^−3^
Control group	A-1 A-2	15°, full anchorage	80.28 79.75	80.02	7.06 7.08	80.28 79.75
Stratification angle	B-1 B-2	30°	88.31 87.71	88.01	7.14 7.10	7.12
	C-1 C-2	45°	102.23 104.71	103.47	7.07 7.32	7.20
Anchoring method	D-1 D-2	End anchorage	67.60 68.69	68.15	6.75 6.55	6.65
	E-1 E-2	No anchorage	61.03 60.07	60.55	6.39 6.31	6.35
